# Improving the efficacy of combined radiotherapy and immunotherapy: focusing on the effects of radiosensitivity

**DOI:** 10.1186/s13014-023-02278-5

**Published:** 2023-05-24

**Authors:** Zhiru Gao, Qian Zhao, Yiyue Xu, Linlin Wang

**Affiliations:** 1 Department of Radiation Oncology, Shandong Cancer Hospital and Institute, Shandong First Medical University and Shandong Academy of Medical Sciences, Jinan, Shandong 250117 China; 2 Department of Oncology, Renmin Hospital of Wuhan University, Wuhan, 430064 China

**Keywords:** Radiotherapy, Immunotherapy, Radiosensitivity, Immune checkpoint blockade, Radiosensitivity index

## Abstract

Cancer treatment is gradually entering an era of precision, with multitude studies in gene testing and immunotherapy. Tumor cells can be recognized and eliminated by the immune system through the expression of tumor-associated antigens, but when the cancer escapes or otherwise suppresses immunity, the balance between cancer cell proliferation and immune-induced cancer cell killing may be interrupted, resulting in tumor proliferation and progression. There has been significant attention to combining conventional cancer therapies (i.e., radiotherapy) with immunotherapy as opposed to treatment alone. The combination of radio-immunotherapy has been demonstrated in both basic research and clinical trials to provide more effective anti-tumor responses. However, the absolute benefits of radio-immunotherapy are dependent on individual characteristics and not all patients can benefit from radio-immunotherapy. At present, there are numerous articles about exploring the optimal models for combination radio-immunotherapy, but the factors affecting the efficacy of the combination, especially with regard to radiosensitivity remain inconclusive. Radiosensitivity is a measure of the response of cells, tissues, or individuals to ionizing radiation, and various studies have shown that the radiosensitivity index (RSI) will be a potential biomarker for predicting the efficacy of combination radio-immunotherapy. The purpose of this review is to focus on the factors that influence and predict the radiosensitivity of tumor cells, and to evaluate the impact and predictive significance of radiosensitivity on the efficacy of radio-immunotherapy combination.

## Introduction

Radiotherapy can provide excellent local control of tumor growth by directly inducing single strand breaks (SSBs) and double strand breaks (DSBs) in DNA, as well as apoptosis and necrosis of tumor cells through the formation of reactive oxygen species (ROS) and free radicals, and is an irreplaceable therapeutic tool in cancer treatment [1]. Radiotherapy also has potent immunomodulatory potential by promoting tumor-specific antigen production and enhancing the initiation and activation of cytotoxic T cells, thereby allowing tumor clearance in immune surveillance. In addition, radiotherapy may induce immunogenic cell death through the release of cytokines, inflammatory mediators, and other immune-related molecules. Despite the activated CD8^+^ T cells and other immunostimulatory cells can migrate and infiltrate to metastatic sites to act as anti-tumor, but the upregulation of immune-suppressed cells by inflammatory factors may inhibit the anti-tumor effects and lead to tumor progression. This suggests that radiotherapy alone is not sufficient to completely eliminate primary and metastatic tumor lesions [2].

Based on the understanding of cytotoxic T lymphocyte-associated antigen 4 (CTLA-4) and the programmed cell death protein 1/ programmed death-ligand 1 (PD-1/PD-L1) and other pathways in tumor immune microenvironment, immune checkpoint inhibitors (ICIs) can enhance the intrinsic immune response against tumor antigens by promoting T cell activation and function, and have been approved for the treatment of a variety of tumors [3]. However, not all patients derive benefit from this treatment and the effective rate of ICIs alone is only 20-30%, with a majority of patients initially developing primary drug resistance or acquiring secondary drug resistance soon after treatment [4]. Augmented immunotherapy involves increased release of tumor antigens, T cell infiltration, and enhanced antigen presentation. Several mechanisms of immune escape have been postulated to explain the failure of tumor immune attacks. A better understanding of these mechanisms will help us to seek therapeutic strategies to overcome immunotherapy resistance [5].

**Fig. 1:**
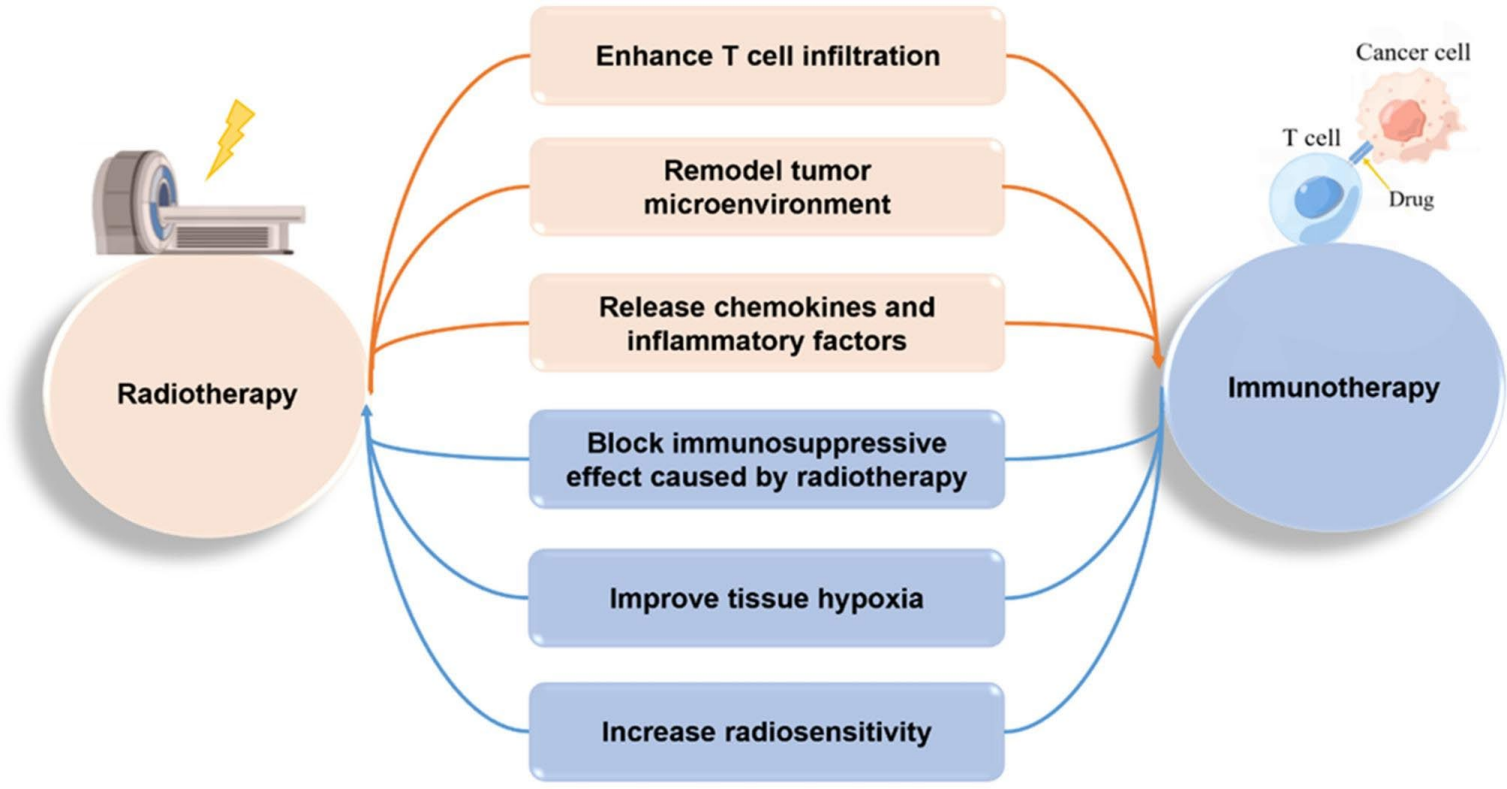
Mechanism of interaction between radiotherapy and immunotherapy

In recent years, the combination of radiotherapy and immunotherapy (CRI) can increase mutual sensitization and enhance antitumor effects, and their synergistic effects have shown survival benefits in multiple studies [6]. To begin with, radiotherapy triggers the release and presentation of tumor-associated antigens (TAAs), which enhance systemic responses by triggering the recruitment of antigen-presenting cells (APCs), such as macrophages, dendritic cells (DCs), and B cells that enhance T-cell infiltration and promote anti-tumor immune responses in the host [7]. Activation of tumor cells by radiotherapy can reshape the tumor microenvironment to reduce immunotherapy resistance, induce antigen release and cross-presentation of DCs, and trigger the recruitment and activation of APCs, which play a key role in the antitumor immune response [8, 9]. Moreover, radiation promotes the release of cytokines and chemokines, which leads to increased production and recruitment of fibroblast growth factor (FGF), transforming growth factor-β (TGF-β), interleukin 1β (IL-1β) and tumor necrosis factor (TNF), which activate Treg cells, bone marrow-derived suppressor cells and cancer-associated fibroblasts [10, 11]. A recent study indicated that radiation-induced DNA DSBs upregulate PD-L1 expression in tumor cells via ATM/ATR/Chk1 kinase, but immunotherapy can prevent the immunosuppressive effects caused by radiotherapy [12]. In addition, dysfunction of the tumor vascular system can lead to an immunosuppressive microenvironment and induce radioresistance, and immunotherapy creates a potential opportunity to reduce tumor hypoxia and improve radiosensitivity. When tumor cells are activated by immunotherapy, activation of CD8^+^ T cells and production of interferon (IFN)-γ to normalize tumor vasculature can sensitize tumors to radiation therapy through mechanisms that include normalization of the tumor vascular system and tissue hypoxia [13–15] (Fig. 1). Therefore, the CRI to synergistically counteract the innate and adaptive immunity of cancer cells, as well as to bypass immune tolerance and exhaustion is highly prospective clinically.

The identification of biomarker-based approaches is central to the development of clinical strategies for CRI, but most of the previous studies on the efficacy of CRI have focused on the dose, timing, efficacy, and sequence of the combination of the two treatments [13, 16]. There is no standardized choice for the sequence of radiotherapy combined with immunology, so the timing used in different studies currently varies. In multiple preclinical and clinical trials, immunology prior to or concurrent with radiotherapy is the superior choice [17, 18]. There is additional evidence to suggest that sequential therapy and the early use of immunotherapy after radiotherapy can increase the clinical benefits, which is beneficial for newly recruited T cells to destroy tumors [19]. Although preclinical works have shown that immunotherapy has a radiosensitizing effect, the window of opportunity for optimizing this synergy is limited as it includes many confounding factors [20]. Therefore, the optimal sequence of radiotherapy and immunotherapy still needs to be explored through large randomized clinical trials. There is now increasing evidence that the intrinsic radiosensitivity in tumor cells also influences the release of cancer cell antigens and affects antigen-specific T cell activation during the radiation-induced cancer immune cycle [21]. As we all know, the most significant radiobiological factors affecting tumor response to radiotherapy are summarized as the “5 Rs”: DNA damage repair, redistribution in cell cycle, repopulation, reoxygenation, and intrinsic radiosensitivity of cancer cells [22]. Among them, the radiosensitivity of tumor cells is the main determinant of tumor response to radiation [23]. Recently, reactivation of the antitumor immune response has been recognized as the “6th R”, which extends the concept of radiosensitivity beyond the tumor cells themselves and supports improved outcomes when radiotherapy is combined with immunotherapy [24]. In this review, we focus on the radiosensitivity of tumor cells to explore its influencing factors, prediction methods and interactions on the immune system. Furthermore, we explore the predictive value of radiosensitivity to CRI efficacy, which is expected to provide new directions for improving the efficacy of CRI.

### The influence factors of tumor radiosensitivity

**Fig. 2:**
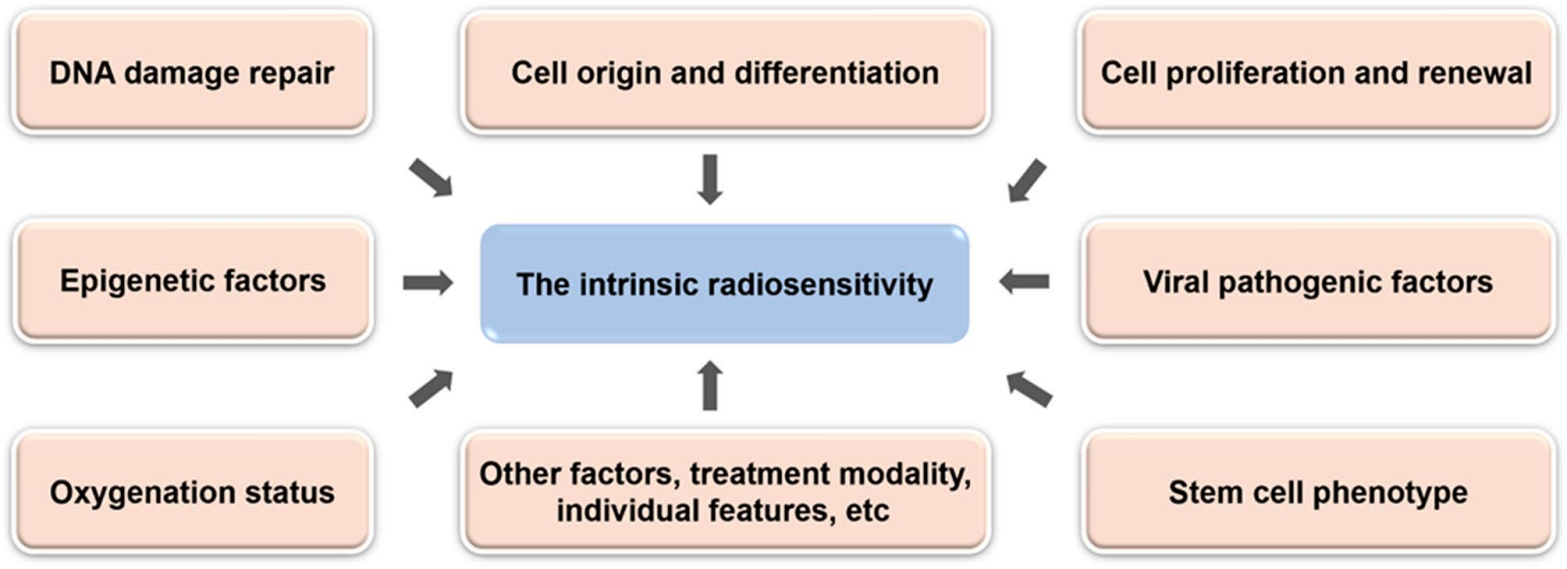
The influence factors of tumor intrinsic radiosensitivity

The intrinsic radiosensitivity of tumor cells is the main determinant of tumor response to radiation, which involves multiple tumor signaling pathways and molecular biological information [23] (Fig. 2). The cellular origin and differentiation of tumor tissues are the main factors affecting the radiosensitivity of tumor cells. Tumors originating from radiosensitive tissues are more sensitive to radiation, while poorly differentiated tumors are less sensitive to radiation [25]. The radiosensitivity of an individual depends to a large extent on biological factors related to epigenetic factors, and the epigenetic mechanisms that determine the selection of metabolic patterns also contribute to the individual radiosensitivity and adaptability of an organism. On the one hand, DNA methylation affects the initial damage process, and on the other hand, methylation shift to ab initio type is associated with further development of protective and repair processes [26]. However, the exact underlying genetic factors that contribute to the inter-individual differences in cellular radiosensitivity are unknown. Understanding the cellular and genetic basis of radiosensitivity and identifying individuals with higher or lower radiosensitivity will facilitate population risk assessment, disease prediction, individualized radiotherapy, and the development of radiation protection standards [27]. Moreover, observations of human tumors have revealed a clear relationship between cell proliferation and cell renewal rates and radiosensitivity. Any tumor with rapidly average growth rate and elevated cell renewal rate is also more sensitive to radiation, and the cellular radiosensitivity differs in different periods, so the redistribution of cell cycle phases within the cell population after irradiation can alter the radiosensitivity.

In spite of the many factors (i.e., dose, exposure volume, gender, age, underlying disease, and lifestyle) that may influence individual radiosensitivity and radiosensitivity to cancer, the inherent cellular radiosensitivity is genetically determined and supported by genetic alterations involving DNA damage repair [28, 29]. Genetic alterations in proteins involved in DNA damage repair are responsible for individual differences in radiation response. Genetic mutations in DNA repair response-related genes (i.e., p53, ATM, BRCA1, BRCA2, ERCC1, XRCC3 and Rad51) have also been found to be associated with radiosensitivity in lung cancer correlation [30, 31]. For instance, individuals with pure mutations in ATM have an approximately three-fold increase in radiosensitivity at the cellular, tissue and biological levels compared to average [32]. The development of DNA-based markers is currently underway, and areas for additional research include the role of somatic mutations in DNA damage response genes that affect radiosensitivity. Exposure of cells to extracellular matrix proteins can increase radioresistance by promoting DNA damage repair and activation of the Akt/MAPK signaling pathway [33]. It has been demonstrated that the anti-apoptotic protein nucleolin (C23) can enhance radiosensitivity in non-small cell lung cancer (NSCLC) by affecting the activity of DNA-dependent protein kinase (DNA-PK) [34].

There is growing evidence that viral pathogenic factors are associated with the regulation of cellular radiation response, treatment outcome, and clinical prognosis in patients following radiotherapy, with the regulation of DNA damage repair mechanisms being the most common point of attack [35]. Malignancies with a viral etiology are more immunogenic, such as human papillomavirus (HPV), Epstein-Barr virus (EBV) and other virus types that are more sensitive to anticancer therapy. One work identified a group of Head and neck squamous carcinoma (HNSCC) that may benefit from CRI and showed a significantly improved prognosis in patients with HPV-positive tumors, attributed to increased intrinsic radiosensitivity and possibly to the modulation of cytotoxic T-cell responses in the tumor microenvironment [35]. Recent study indicated that for HPV-positive HNSCC, the virus hijacked cellular mechanisms of DNA repair, altered cell cycle distribution, induced cell proliferation and displayed peculiar hypoxic kinetics during radiation treatment [36]. The mechanism described involves a reduced ability to repair DNA double-strand breaks, accompanied by enhanced radiation-induced G2/M cell cycle arrest [37, 38]. Additionally, excessive expression of immune checkpoints was also strongly associated with radiosensitivity. This finding suggested that high PD-1 expression was significantly associated with the clinical prognosis of HPV/p16-positive HNSCC. Patients in the radioresistant group and HPV/p16-negative group with radioresistant genetic markers could benefit from combination CRI [39]. The central research on EBV-regulated radiation response has focused on LMP-1, which is expressed in most EBV-associated malignancies.LMP-1 inhibits DNA double-strand break repair by inhibiting the phosphorylation and activity of DNA-PKcs, a key enzyme of the NHEJ pathway in nasopharyngeal carcinoma (NPC), and by inhibiting ATM repair of DNA double-strand breaks [40].

In addition to the tumor cells themselves, environmental factors such as oxygenation status may also affect radiosensitivity by further modulating damage induction and cellular responses [41]. Therefore, as a classical regulator of tumor radiation resistance, the elimination of hypoxia may be a potential solution to address radioresistance [42]. Hypoxia inducible factor-1 (HIF-1) remains active in cells that survive radiation therapy and is associated with tumor cell resistance to radiotherapy. It has been suggested that it may modulate tumor radioresistance through reprogramming of glucose metabolism and cell cycle regulation [43]. Tumors contain different proportions of intrinsically radioresistant cancer stem cell (CSC), which are closely associated with tumor hypoxia, and HIF-1α contributes to the development and maintenance of the CSC phenotype [44]. The radioresistance of CSC is characterized by a reduced accumulation of radiation-induced DNA damage and increased activation of anti-apoptotic signaling pathways compared to differentiated tumor cells [45]. Current strategies for predicting normal tissue radiosensitivity are genomics and large-scale prospective studies, and further research is still needed to explore the best predictive methods for radiosensitivity [46].

### The prediction methods of tumor radiosensitivity

The radiosensitivity of tumor cells is strongly influenced by molecular variation at the genomic, transcriptional and translational levels. Radiosensitivity is a measure of the response of cells, tissues or individuals to ionizing radiation and can be used to predict which individuals will benefit from radiotherapy. Recent advances in gene sequencing technology and microarray technology for high-throughput RNA analysis have driven interest in identifying features that measure the intrinsic radiosensitivity of tumor cells. The development of a successful predictive analysis of radiosensitivity has been a major goal of research, and many genetic markers have been developed to predict the radiosensitivity of tumors [47]. These methods can be broadly divided into two categories: one is the characterization of the surviving fraction of cancer cell lines formed after radiation, which reflects the intrinsic radiosensitivity of cancer cells, but fails to consider the influence of non-malignant cells in the tumor microenvironment, particularly the role of anti-tumor immunity [48]. The second is the prediction of patient progression after radiotherapy. This is dedicated to predicting the clinical outcome of radiotherapy, but cannot be used for cellular level studies and it is difficult to reveal radiobiologically based mechanisms [49]. Nevertheless, how to build a radiosensitivity prediction model has not been discussed systematically in these recent years.

The traditional experimental approach to determine intrinsic radiosensitivity is the survival of tumor cell lines at a single dose of 2 Gy (SF2), but is not applicable for routine use and alternative strategies must be sought. The radiosensitivity index (RSI) is a 10-gene model based on the survival of 48 human cancer cell lines at SF2 radiation and is a measure of clonogenic survival after a given radiation dose [50]. The 10-gene model (AR, cJun, STAT1, PKC, RelA, cABL, SUMO1, CDK1, HDAC1, and IRF1) that hold a crucial role in DNA damage response, histone deacetylation, cell cycle regulation, apoptosis and proliferation [50, 51]. The RSI prediction model is a linear regression algorithm and is independent of the cancer type. RSI is designed to detect intrinsic tumor radiosensitivity independently of cancer type and has been independently validated as a pan-tissue biomarker of radiosensitivity at multiple disease sites [52–54]. The 31-genes were developed by analyzing a panel of NCI-60 cancer cells that were associated with SF2 expression, and its correlation with radiosensitivity has been validated in various malignancies [48]. Similarly, measuring the oxygen partial pressure of a tumor can indicate its level of hypoxia, which can help predict its radiosensitivity [23]. Unfortunately, these parameters, even when used in combination, are insufficient to predict tumor radioresistance for clinical use.

Since the relationship between radiation dose and survival is nonlinear, various mathematical formulas have been proposed to fit the radiation survival curve. The linear quadratic (LQ) model has become the most popular calculator for analyzing and predicting ionizing radiation response in the laboratory and in the clinic, where the α/β ratio is used to characterize the sensitivity of specific tissue types to segmentation [55]. The LQ model provides a simple equation between cell survival and delivered dose: S = exp (-αD-βD^2^) [56]. The radiosensitivity of cells is influenced by complex interactions between intrinsic polygenic traits. As the mechanisms and biomarkers of radiosensitivity have become better understood, gene expression classifiers containing few key genes have been used to predict radiosensitivity in specific tumor types or various human cancers [57, 58]. Based on RSI, LQ model, and the time and dose of radiotherapy received by each patient, a team derived a genome-based model for adjusting radiotherapy dose (GARD) on more than 8,000 tumor samples from more than 20 tumor types [59]. The GARD predicts the efficacy of radiotherapy and guides the radiation dose to match the individual tumor radiosensitivity, with higher GARD values associated with better efficacy of radiotherapy. Given that the range of GARD values varies among different types of cancer, the use of RSI alone cannot be a complete representation of the treatment effect, and we need to combine the means of tumor type and genetic testing to determine the appropriate radiotherapy dose for individual patients.

**Fig. 3:**
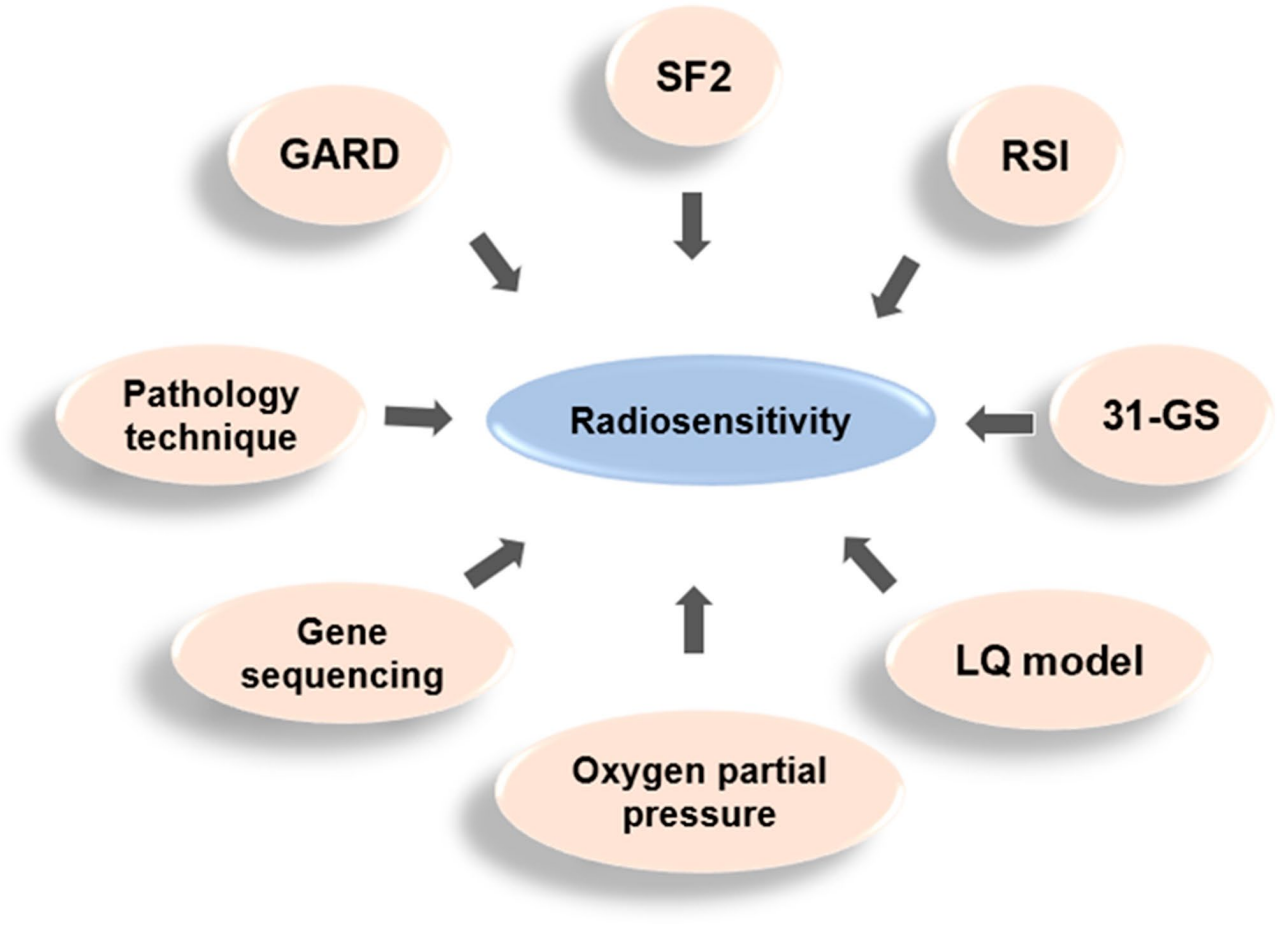
Common prediction methods for radiosensitivity

Besides the classical biological mechanisms mentioned above, gene sequencing has further revealed the regulatory role of non-coding RNAs on radiosensitivity, and their high-throughput properties contribute to the study of radiosensitivity mechanisms. A previous study used a gene expression classifier to predict radiosensitivity, which regarded radiosensitivity as a continuous variable, used microarray significance analysis for gene selection, and multiple linear regression model for radiosensitivity prediction [57]. Three new genes (RbAp48, RGS19 and R5PIA) were identified in the gene selection step, and their expression values were correlated with radiosensitivity and were transfected with cancer cell lines. The results established that the RbAp48 gene could induce radiosensitivity 1.5-2 times, and increased the proportion of cells in G2-M phase of cell cycle. In addition, the study also showed that the overexpression of RbAp48 was related to the dephosphorylation of Akt, which suggested that RbAp48 may exert its effects by antagonizing the Ras pathway. This study established that radiosensitivity can be predicted based on gene expression profiles and introduced a genomic approach to identify novel molecular markers of radiosensitivity [60]. Moreover, some traditional pathology techniques remain valid for measuring tumor radiosensitivity. For instance, hematoxylin and eosin staining can be used to identify radiosensitive (i.e., seminoma) or radioresistant (i.e., glioma) tumors [61] (Fig. 3). More advanced pathologic techniques such as DNA methylationome analysis are now used to classify tumors, but have not yet guided the clinical prescription of radiotherapy doses. The current strategy for predicting normal tissue radiosensitivity is genomics and large-scale prospective studies, and further studies are still needed to explore the best predictive methods for radiosensitivity.

### The biomarkers of tumor radiosensitivity

Unsatisfactory radiosensitivity has been plagued, and finding biomarkers that predict radiosensitivity could help improve the efficacy of radiotherapy. Chromosomal aberrations and DNA damage, in particular DSB, are among the few cellular markers that have some correlation with cellular radiosensitivity. Signaling pathway molecules involved in the DNA damage response are excellent candidates for the evaluation of radiosensitivity biomarkers, and relevant biomarkers include MRE11, AIMP3, NBN, and BRE, with MRE11 potentially a predictive biomarker for radiotherapy benefit [62]. The current research suggests that γ-H2AX assay as a rapid and sensitive biomarker can be used in epidemiological studies to measure changes in radiosensitivity. The use of γ-H2AX lesion analysis as well as DSB repair gene polymorphisms can be used to assess cellular radiosensitivity, which will assist in population risk assessment, disease prediction, individualized radiotherapy, and the development of radiation protection standards [63]. Additionally, evaluation of the predictive significance of the systemic immune-inflammatory index (SII) on overall survival and radiosensitivity in advanced NSCLC showed favorable radiosensitivity in the low SII group, and higher SII levels were associated with poorer overall survival and radiosensitivity [64]. Cellular radiosensitivity can be assessed by quantifying DSB damage and repair [65]. It has been observed that among the different types of DNA damage, DSBs have the slower and most lethal repair dynamics. Therefore, they are more helpful in explaining clinical radiosensitivity than other types of damage with rapid repair dynamics [66]. The development of DNA-based markers is currently underway and areas for further research include the role of somatic mutations in DNA damage response genes that affect radiosensitivity [67].

The molecular mechanisms involved in the radiation-induced response are complex and the expression levels of genes do not consistently represent the properties of all proteins in the tumor cells. The proteomic approach allows the identification of various proteins involved in the cellular response to ionizing radiation, which may be useful in identifying potential candidates for use as predictive biomarkers. The expression levels of genes do inconsistently represent the nature of all proteins in normal or tumor cells, and therefore direct detection of protein expression may be more effective in determining the complexity of the mechanisms and the large number of molecular signatures involved in the cellular radiation-induced responses. The radiosensitivity of tumors is related to the basal expression levels of intracellular or cell membrane proteins, and the direct detection of protein expression using proteomics studies allows the detection of protein sequences and post-translational modifications stored in genes that can be used for early diagnosis, prognosis and treatment of cancer [68]. Current proteomics technologies can be used to detect and analyze proteomic information using cells, tissues or body fluids, providing a better platform for biomarker research and development [69, 70]. High throughput radio proteomics is the latest tool, where mass spectrometry (MS) is used to analyze and identify unknown proteins by converting protein molecules into gas phase ions through an ionisation source and applying the electromagnetic field of the instrument to separate proteins with a specific mass-to-charge ratio. MS has the advantage of quickly analysis, high sensitivity and resolution. The advantages of MS are its speed, sensitivity and resolution. Proteomics research based on liquid chromatography-mass spectrometry (LC-MS) is now widely used [71]. The intrinsic radiosensitivity of NSCLC is mainly regulated by the signal pathways in the proteoglycans, focal adhesion and the actin cytoskeleton in cancer. Radiosensitivity-specific proteins can guide clinical individualized radiotherapy by predicting radiation response in NSCLC patients [72].

### The effect of radiosensitivity on the efficacy of CRI

In the era of immunotherapy, reliable genomic predictors to identify optimal patient populations in CRI are lacking. A comprehensive analysis of radiosensitivity-associated genes and proteins in lung cancer and other solid tumors has been used to identify potential biological predictors of radiosensitivity [73]. There are some evidences that radiosensitivity could predict the effect of radiotherapy and immunotherapy **(**Table 1**)**. To determine first whether tumor radiosensitivity correlates with immune system activation in all tumor types, Tobin et al. identified 10,240 genotypically distinct solid primary tumors using 12 chemokine genes to define intratumor immune activation and determined that low RSI was significantly associated with elevated immune activation, supporting the association of RSI with immune-related signaling networks in patients’ tumors (using an RSI threshold of 0.3745) [74]. In another study, a total of 12,832 primary tumors from 11 major cancer types were analyzed in relation to DNA repair and immune subtypes in order to determine whether genomic scores of radiosensitivity were associated with immune responses. The results found that RSI was related with various immune-related signatures and predicted responses to PD-1 blockade, emphasizing the promising potential of RSI as a candidate biomarker for CRI [75]. In addition, a study also identified enhanced immune checkpoint interactions in radioresistant tumors, providing a new theoretical basis for radiotherapy and ICIs for the treatment of HNSCC [76]. The RSI-low may be characterized by higher genomic instability and subsequently higher mutational burden, which associated with predicted efficacy of dominant IFN-γ signaling responses and PD-1 blockade. Taken together, RSI-Low tumors may represent a special subgroup and therapeutic target for immunotherapy [75].

**Table 1:** The predictive role of radiosensitivity in radiotherapy and immunotherapy

Study	Cancer type	Sample Size(n)	Outcome
Tobin et al. (2017) [74]	Breast cancer	282	RSI-low status that refer to more radiosensitive tumor (HR 0.58, 95% CI 0.34-1.00; *p* = 0.05) and 12-CK-high status that refer to more immune-active tumor (HR 0.61,95% CI 0.39–0.96; *p* = 0.03) were independently related with improved distant metastasis free survival.
Dai et al. (2021) [75]	11 major cancer types	12,832 primary tumors and 585 metastatic tissues	RSI was significantly associated with immune-related molecular features(*p* < 0,05). RSI-Low tumors carried more higher portion of follicular T helper cells, T cell gamma delta cells, activated NK cells and M1 macrophages than RSI-High tumors.
Grass et al. (2022) [87]	31 primary tumors types	10,469	A weak negative relevance between the RSI and immune score (Pearson’s r = − 0.28; Spearman’s r = − 0.27, *P* < 0.001). Tumors with high radiosensitivity showed significant enrichment of IFN-related signaling pathways and immune cell infiltration (i.e., CD8^+^ T cells, activated NK cells, M1-macrophages, q < 0.05).

RSI, radiosensitivity index; NK, natural killer; IFN, interferon

The molecular mechanisms underlying the biological effects of radiotherapy can affect the response and repair of cells to DSBs, but there is currently limited research on the mechanisms of RSI and immune response [77].Research has found that RSI is associated with various immune-related genomic and molecular characteristics, and low RSI is correlated with dominant response to IFN-γ signaling and predicted efficacy of PD-1 blocking agents [75]. Lower RSI is linked to higher HRD scores and higher TMB, indicating the presence of defective DNA repair mechanisms and potential for response to immune based therapies [78]. Besides, lower RSI is also correlated with higher RNA stemness score, indicating higher degrees of stemness and tumor de-differentiation, which is also related to increased PD-L1 protein expression [79].To further explore the relationship between RSI and immune response, a team used whole transcriptomic and matched proteomic data from 12,832 primary and 585 metastatic tumors and found that RSI was associated with a variety of immune-related genomic and molecular features. Lower RSI was associated with higher homologous recombination deficiency (HRD) scores and higher tumor mutational burden (TMB), suggesting the presence of defective DNA repair mechanisms and response potential to immune-based therapies [78]. HRD scores were correlated with genes involved in homologous repair, including BRCA1, BRCA2, RAD51B, and RAD51C, and alterations in these genes were related to radiosensitivity [80, 81]. Intriguingly, the RSI-Low tumors exhibit both higher microsatellite instability (MSI) and TMB molecular profiles in gastric cancer, which were shown to be subgroups with favorable prognosis after immunotherapy [82]. Furthermore, since RSI genes (STAT1 and IRF1) are downstream of IFN-γ-mediated signaling, RSI correlates better with various immune-related molecular features and phenotypes than other genes and genetic features associated with radiation response [83].

At the same time, the role of the immune system is crucial for tumor radiosensitivity. To explore the relationship between intrinsic tumor radiosensitivity and the immune system, a study has investigated radiation-induced tumor equilibrium and dormancy in animal models, and whether host immune responses contribute to radiation-induced tumor equilibrium [84, 85]. The study developed two mouse models—TUBO (HER2-positive breast cancer) and B16 (melanoma), and has observed four possible tumor responses to radiotherapy. These were non-responsive tumors (non-responsive to radiotherapy); responsive tumors (tumor regression observed within 10 days after radiation); stable tumors (tumors that regress and remain stable and palpable during any 34-60-day observation period); late recurrent tumors (tumor recurrence after 60 days). The inherent cellular radiosensitivity of tumors is frequently hypothesized to explain the observed differences in tumor regeneration rates observed after radiotherapy, and this study determined the radiosensitivity of tumor cells taken from mice that responded variably to radiotherapy. These tumors were surgically removed and digested into single cell suspensions and subjected to 2, 5, or 10 Gy of in vitro irradiation and assessed with clonogenic assays. The results demonstrated that tumor cells with different responses to radiation in vivo exhibited indistinguishable radiosensitivity in vitro. This finding revealed that the degree of tumor cells radiosensitivity was unable to explain the different tumor responses to local radiotherapy, in contrast to immune cells and their cytokines, which have been shown to exhibit a pivotal role in inhibiting tumor cell regeneration in two experimental animal model systems.

Traditional radiosensitivity studies have focused on tumor cells, neglecting the effects of the tumor microenvironment, which consists of stromal and immune cells [86]. To explore the relationship between RSI and its associated unique tumor immune microenvironment, a study used RSI to assess the radiosensitivity of 10,469 primary tumor samples and to assess the immune environmental components of each tumor. The results showed that tumors with high immune cell content were more sensitive to radiation because they were enriched with leukocytes, which are highly sensitive to radiation. Furthermore, tumors estimated to be highly sensitive to radiotherapy exhibited significant enrichment of interferon-related signaling pathways and immune cell infiltration (i.e., CD8^+^ T cells, activated natural killer cells, M1 macrophages) [87]. In the radiation-induced cancer immune cycle, intrinsic radiosensitivity affects cancer cell antigen release and immune status affects antigen-specific T cell activation [88]. To elucidate the effect of tumor microenvironment on the efficacy of radiotherapy in glioma patients, a study analyzed the differences in the infiltration levels of immune cells. Patients were classified into a radiosensitive (RS) group and a radioresistant (RR) group. The results showed that the level of activated NK cell infiltration was significantly higher in the RS group, whereas the level of macrophage, Treg cell, and resting NK cell infiltration was significantly higher in the RR group, and the immune score and PD-L1 expression levels were significantly higher in the RR group than in the RS group. These results indicated that patients in the RR group had higher immunogenicity, higher TMB and mutational characteristics, which requires more clinical trials to demonstrate [89, 90].

### Integrating tumor radiosensitivity and immune status to predict clinical outcomes

In addition to focusing only on intrinsic tumor radiosensitivity, the integration of radiosensitivity features and immune features could predict the clinical outcomes of patients **(**Table 2**)**. One study has developed independent predictors of radiosensitivity signature (RSS) and an immune signature (IMS) in breast cancer patients treated with radiotherapy. When integrating both signatures, patients with radiosensitive or immune effective tumors gained better disease-specific survival (DSS) from radiotherapy. On the contrary, patients in the other group, defined as radiotherapy resistance and immunodeficient, had significantly lower DSS when they received radiotherapy. Individuals in the radiosensitive and immunodeficient or radiotherapy resistant and immune effective, there was no significant difference in DSS between treatment groups [91]. Another study in the Cancer Genome Atlas (TCGA) dataset showed significantly higher PD-L1 expression in the RR group than in the RS group, and the PD-L1-high-RR group had the worst survival, so the analysis focused on this group of patients. These studies demonstrated that 31 genetic features and PD-L1 expression status as potential predictive markers for radiotherapy. Moreover, patients classified as PD-L1-high-RR exhibit radiotherapy resistance and immunosuppressive TME through multiple mechanisms and may benefit from radiotherapy combined with PD-1/PD-L1 blockers. Therefore, the integration of 31 genetic characteristics and PD-L1 expression status may help to classify the patient population that may benefit most from the combination of radiotherapy and PD-1/PD-L1 blockade in clinical practice [92]. In addition, it has been shown that RSI and PD-L1 status predict clinical outcome in patients with glioblastoma multiforme. The 399 patients were divided into RS and RR groups based on radiosensitivity genetic markers and into PD-L1 high and PD-L1 low groups based on CD274 mRNA expression. Differential and comprehensive analyzes of expression and methylation data were performed. The results demonstrate the potential efficacy of radiotherapy in combination with PD-1/PD-L1 blockade and angiogenesis inhibition in the PD-L1-high-RR group [93].

**Table 2:** The predictive role of radiosensitivity and immune gene signatures for clinical outcome of patients

Study	Cancer type	Sample Size(n)	Outcome
Cui et al. (2018) [91]	Breast cancer	1439	Patients treated with radiotherapy had significantly better DSS in the immune-effective group (HR 0.46; *P* = 0.0076). Both radiosensitivity and immune signatures could predict the benefit from radiotherapy (P_interaction_=0.007 and 0.005).
Jang et al. (2018) [92]	Lower grade glioma	511	Patients classified as the PD-L1-high-radioresistant group showed a detrimental effect on OS rate and may benefit most from radiotherapy combined with immunotherapy (HR: 1.96; CI: 1.01–3.80; *p* = 0.047).
Jang et al. (2020) [93]	Glioblastoma	399	PD-L1-high-radioresistant group could potentially benefit from radiotherapy combined with immunotherapy and angiogenesis inhibition (HR, 1.70, 95%CI, 1.03–2.81; *p* = 0.037).
Dai et al. (2021) [94]	Head and neck squamous cell carcinoma	288	The survival rate and B cell count of the radioresistant and PD-L1-high group were lower than those of the other groups (*p* < 0.05).
Sun et al. (2021) [97]	Head and neck squamous cell carcinoma	392	Only patients in the radiosensitive-immune group had better OS after receiving radiotherapy (HR 0.194, 95%CI 0.788–0.480; *p* < 0.001).

DSS, disease specific survival; OS, overall survival

Tumor radiosensitivity is also governed by other features of cancer, including tumor microenvironment dynamics, nutrient utilization, and multiple cellular complexes. A study showed that the RR-PD-L1-high group had depleted B cells and had a significantly lower survival rate than the other groups, which predicted the prognosis of patients with locally advanced HNSCC [94]. Some evidence points to the possibility that the pathways associated with radiosensitivity may also modulate the immunogenicity of tumors and predict their response to immunotherapy. For example, inactivation of the DNA repair mechanism may trigger an immune response and impair tumor growth by triggering the release of neoantigens, and the therapeutic efficacy of immunotherapy can be predicted by the presence of these DNA repair defects [95]. Several common regulators of DNA repair and immune checkpoints have been identified, such as PARP inhibitors capable of DNA repair proficiency and radiosensitization of tumor cells [96]. Several studies have demonstrated that the combined stratification of intrinsic radiosensitivity and immune status is superior to considering intrinsic radiosensitivity or immune status separately, and can therefore be used in preclinical evaluations to select patients or to determine whether radiation sensitizers and immunotherapy should be used together [97]. With respect to whether immunotherapy modulates tumor intrinsic radiosensitivity, increasing evidence supports the idea that DNA repair defects modulate tumor immune checkpoints, but whether the immune checkpoints in turn modulate DNA repair pathways remains unclear, and this potential new mechanism by which immunotherapy modulates tumor intrinsic radiosensitivity still deserves further exploration in the future.

### Future Prospect

Since intrinsic radiosensitivity and immune status affect the initial and effective phases of the radiation-induced cancer immune cycle, respectively, it is necessary to consider radiation in combination with immunity when selecting patients who may benefit from radiotherapy. Moreover, the prognostic value of RSI has been validated using multiple independent datasets, such as those used to predict the prognosis of patients treated with radiation for breast, pancreatic, glioblastoma, esophageal, and metastatic colorectal cancers [51, 98–100]. Despite the recognized differences in tumor radiosensitivity in preclinical and clinical settings, radiation dose prescriptions are not currently individualized in the field of radiation oncology based on the biology of the patient’s tumor. However, individualized adjustment of radiation dose based on patient tumor radiosensitivity is a promising strategy for effective radiotherapy, and radiosensitivity indices are expected to be potential biomarkers for combination radiotherapy and immunotherapy.

## Conclusion

In this review, we first present the mechanisms underlying the interaction between radiotherapy and immunotherapy, where radiotherapy serves as an essential adjunct to immunotherapy by providing a source of danger signals, antigens and activation of innate immunity. Similarly, immunotherapy can sensitize tumors to subsequent radiotherapy, reducing the radiation dose required to eradicate them. We next describe the effect of tumor cell radiosensitivity and the method to predict it. The biological effects of radiation are mediated by a complex network of signaling pathways, and advances in genomics can be used to guide radiotherapy alone or in combination, and the commercialization of genomic-based tools will be important to facilitate its implementation. Furthermore, radiosensitivity holds favorable promise for the predictive role and clinical application of radiation-free combination, and future clinical investigations will need to emphasize the implementation of preclinical and translational discovery data in the development of new clinical trials to demonstrate reproducibility in the patient setting and to help optimize the efficacy of their combination therapy. In summary, the radiosensitivity of tumor cells can help predict the efficacy of CRI and the integration of immune status with radiosensitivity can also help better predict clinical outcome. In the future, the treatment of CRI should rely on the mining and detection of multiple biomarkers to achieve precision oncology.

## Data Availability

Not applicable.

## Abbreviations

APCs: Antigen-presenting cells
CRI: Combination of radiotherapy and immunotherapy
CSC: Cancer stem cell
CTLA-4: Cytotoxic T lymphocyte-associated antigen 4
DCs: Dendritic cells
DNA-PK: DNA-dependent protein kinase
DSBs: Double strand breaks
DSS: Disease-specific survival
FGF: Fibroblast growth factor
GARD: Adjusting radiotherapy dose
HIF-1: Hypoxia inducible factor-1
HNSCC: Head and neck squamous carcinoma
HPV: Human papillomavirus
HRD: Homologous recombination deficiency
ICIs: Immune checkpoint inhibitors
IFN: Interferon
IL-1β: Interleukin 1β
IMS: Immune signature
LC-MS: Liquid chromatography-mass spectrometry
LQ: Linear quadratic
MS: Mass spectrometry
MSI: Microsatellite instability
NK: Natural killer
NPC: Nasopharyngeal carcinoma
NSCLC: Non-small cell lung cancer
PD-1: Programmed death protein-1
PD-L1: Programmed death-ligand 1
ROS: Reactive oxygen species
RR: Radioresistant
RS: Radiosensitive
RSI: Radiosensitivity index
RSS: Radiosensitivity signature
SII: Systemic immune-inflammatory index
SSBs: Single strand breaks
TAAs: Tumor-associated antigens
TCGA: The Cancer Genome Atlas
TGF-β: Transforming growth factor-β
TMB: Tumor mutational burden
TNF: Tumor necrosis factor
